# Doctors' experiences of earlier mandatory postgraduate communication skills training: a qualitative study

**DOI:** 10.5116/ijme.6213.7ee7

**Published:** 2022-02-28

**Authors:** Jane Ege Møller, Jakob Henriksen, Charlotte Søjnæs, Matilde Nisbeth Brøgger

**Affiliations:** 1 Department of Clinical Medicine, HEALTH, Aarhus University, Denmark; 2 Department of Clinical Pharmacology, Aarhus University Hospital, Denmark; 3 Copenhagen Academy for Medical Education and Simulation (CAMES), Centre for Human Resources and Education, The Capital Region of Denmark, Denmark; 4 Center for Health Communication, School of Communication and Culture, ARTS, Aarhus University, Denmark

**Keywords:** Communication skills, postgraduate training, resident, doctor, qualitative

## Abstract

**Objectives:**

This study explored the question: what are doctors' perspectives on and experiences with their earlier mandatory postgraduate communication skills training?

**Methods:**

The study used a qualitative, exploratory design. We used purposeful sampling based on the principle of maximal variation to ensure different clinical perspectives. Thus, three focus groups were formed with 12 doctors who had attended mandatory postgraduate communication skills training within 1-9 years prior to the study. The doctors were from three specialties: internal medicine, oncology, and general practice. We used a semi-structured interview guide, and the focus groups were video-recorded. Thematic analysis was used to analyze the data material. Through an iterative process, we identified main and sub-themes.

**Results:**

The first-year residency mandatory postgraduate communication skills training provided all participants with skills that had helped them in their ongoing clinical work. In addition, five dominant themes were observed: modes of use, the timing of course, experience with experiential methods, sharing challenges with peers, and need for continuous feedback and follow-up.

**Conclusions:**

Doctors value early mandatory postgraduate communication skills training even years after attending the course and request similar ongoing initiatives. Their experiences are positive, they found the timing relevant, and they used the learned skills in their ongoing clinical work, even years after the initial course. Our study indicates that more attention should be given to 'early career' postgraduate communication skills training that is tailored to specific clinical contexts, including hospital settings.

## Introduction

Medical communication skills training is now an integral part of most undergraduate medical schools' curricula. However, except in select specialties, it is rarely systematically incorporated into postgraduate training.^1^^,^^2^ Lack of follow-up training is problematic as research has documented that while clinical communication skills are indeed teachable,^3^^-^^5^ retaining such skills over time can be challenging.^2^^,^^5^^,^^6^ Several studies, therefore, recommend this undergraduate training be supplemented by further training, incorporated within continuing postgraduate medical education.^7^^,^^8^ It has been pointed out that this would facilitate the maintenance and use of such skills in clinical work.^9^

While several studies have investigated medical students' and residents' perspectives on communications skills training,^10^^-^^17^ it is rarely explored how doctors, later on in their medical careers, look back on such training (though one exception is Grant & Hawken 2009).^18^ As stated by Bylund and colleagues. "most evaluation research on communication skills education examines only short-term results".^19^ To our knowledge, only one study has explored the impact of postgraduate communication skills training on doctors' behavior over time.  This study examined whether the time since course completion was related to reported use of communication skills learned during the course.^19^ Their results suggest that the impact of the course was sustained over time, with most physicians (92%) being able to name something specific that they had learned from the course and were currently implementing in their practice. Furthermore, the authors point out that knowledge is needed about how doctors, at different levels in their careers, experience postgraduate communication training. They suggest that future qualitative research should explore this topic. Such qualitative research on long-term perspectives would yield knowledge about not only whether they use it but also how, when, and why it is used. Our study is a contribution to this knowledge gap.

Compared to other countries, Denmark provides a unique case to explore doctors' long-term perspectives on communication skills training as postgraduate communication skills courses have been mandatory since 2004. In addition to the undergraduate communication skills programs, all first-year residents must attend a mandatory three-day communication skills course that focuses on doctor-patient communication (Appendix). Further residency communication training is a workplace-based part of specialty competency training. Only a few specialties include mandatory formal training. Denmark thus provides an obvious context to add knowledge about the experienced usefulness of mandatory postgraduate/resident communication skills training and the long-term usefulness of the training at this level. This study aimed to answer the following research question: What are doctors' perspectives on and experiences with their earlier mandatory postgraduate communication skills training?

## Methods

### Study design and participants

The conceptual framework of the study is 'contextualist', which considers the way people form meaning from their experience and the way the social context influences those meanings.^20^ As the purpose of this study was to investigate perceptions, as opposed to, for example, an observational study examining whether certain skills are indeed used, we adopted an inductive approach to explore thoughts, feelings, and experiences. In line with this, a qualitative design was chosen, consisting of focus groups with doctors who had completed the mandatory communication skills course. The focus group method enables group processes, leading to multi-layered perspectives on a focused topic.^21^ It provides both "the occasion and the stimulus for group members to articulate normally unarticulated normative assumptions".^22^ Within focus groups, the researcher can ask participants to compare their experiences and views, instead of trying to compare individual data from different interviews and speculating on whether or why the interviews and the interviewees' attitudes differ.^23^

We used a semi-structured interview guide, in which discussion topics were organized according to the funnel model.^23^ This starts with a few open questions to enable the participants to speak from their own experiences, followed by more specific questions as well as focused follow-up questions. The main topics presented to the participants were: perspectives on the communication course; personal perspectives on communication; the institutional perspective on communication in their department; how communication is implemented in the workplace; and perspectives on the need for further communication training.

We used purposeful sampling,^24^ with two main recruitment criteria: 1) participants had to have completed the first year of residency and thus have completed the mandatory communication skills training, and 2) participants had to have taken the course at least one year prior to the study. We aimed for data saturation, i.e., the identification of redundancy in the data.^25^ To provide variation in different clinical perspectives, participants were selected from three different specialties: internal medicine, oncology, and general practice. These specialties were chosen to reflect specialties with different communicative tasks ranging from basic patient communication to more complex conversations such as breaking bad news. Participants were informed about confidentiality and participant anonymity. In addition, written informed consent to video-record the interviews were obtained from all participating doctors. The study was approved by the Danish Data Protection Agency. In line with the Consolidation Act on Research Ethics Review of Health Research Projects, approval was not required from the Central Denmark Region Committee on Health Research Ethics.

### Setting

The specific context was doctors who had completed the course in the Central Denmark Region. In this region, approximately 300 first-year residents complete the course each year, and their immediate course evaluation is generally positive (see Appendix, Evaluation). Participants were invited to participate via a colleague. Two of the focus groups were held in the hospital setting, and the third was held in connection with a meeting. The first and second authors conducted the interviews. Both authors are current trainers of the communication course, and the first author also taught the course at the time of the participants' attendance. The focus groups were video-recorded and transcribed verbatim by a research assistant.

In total, 13 medical doctors (4 men, 9 women) participated (see Table 1). The time span since they attended the course ranged from 1-9 years, which allowed for insights into different levels of postgraduate medical experience.

### Data analysis

Thematic analysis was employed as this method enables the identification and analysis of themes in texts.^20^ All authors read and re-read the material. The first and second authors

made initial codes individually guided by the research question. Then, codes were compared, and themes were discussed. All authors reviewed the themes and checked that they were meaningful in relation to both the coded extracts and the full data set, and adjustments were made. All quotations have been translated from Danish.

**Table 1:** Participants' characteristics

Variables		Number
Gender		
	Female (F)	9
	Male (M)	4
Clinical specialty		
	General Practice (GP)	4
	Internal Medicine (IM)	5
	Oncology (O)	4
Years since attending the mandatory communication skills course		
	<2years	2
	2-4years	8
	>4years	3

## Results

A key finding was that all participants valued the course and found that the postgraduate communication skills training provided them with skills that had helped them throughout their years in clinical work. Furthermore, five dominant themes were observed in the material: 1) modes of use, 2) timing of course, 3) experience with experiential methods, 4) sharing challenges with peers, and 5) need for continuous feedback and follow-up.

### Modes of use

Everyone stated that the communication skills they learned during the course were highly useful throughout their professional training and in their clinical work, and that they remembered these skills and applied them in the years after the course. This is expressed in this quotation:

"It's just like a toolkit in the back of your mind that you carry with you every day to work". (I3, F,O,>4Y)

The doctors consciously used the communication skills previously acquired in training, in their continuing work. However different modes of use were displayed. Some stated that they had fully integrated and implemented these communication skills into their clinical work, as illustrated in this example:

"Well, I'm pretty conscious about it actually, relatively often or actually all of the time, really, and I use it a lot to find out what kind of conversation I am about to have." (I5, M, IM, 2-4Y)

Other participants stated that they were not always consciously using the skills but that challenging situations prompted awareness of how to actively use certain communication skills in specific ways. This was evident in situations when the doctors knew in advance that a challenging situation would occur, for example, when breaking bad news, or when unpredictable reactions from patients made the conversation challenging:

"I don't think about it all the time […] but I do reflect on it when these kinds of challenging situations emerge, where you know in advance that it is a difficult and challenging conversation, for example, something that is difficult to explain." (I4, F, GP, 2-4Y)

"If you feel that the patient turns kind of stiff or starts asking questions where you think 'I just answered that'. Or when something is just not like it is supposed to be. Then I think about it [communication]." (I2, F, IM, 2-4Y)

In this way, the skills acquired by the doctor during the course were consciously at play, both when they prepared for what they expected to be a complex or challenging communication, and when unexpected challenges occurred that caught them off guard. Furthermore, they noted their use of communication skills 'when I notice that I start to get annoyed with the patient' as their acquired communicative competency would enable them to try alternative strategies and somehow 'rescue' the communication from ending in an undesirable conflict. Some participants expressed that even though they were only conscious of using communication skills in specific situations, they had a feeling they were using these skills unconsciously all the time:

"Well, like the others are saying, I think more about it in the difficult situations, but to some degree, I always think about it, because if the communication is good, it creates trust […]. You always use it in some way." (I1, F, IM, <2)

### Timing of course

All the participants appreciated that the training took place at the postgraduate level. They expressed that their clinical experience made it more relevant than the undergraduate training they had had during medical school:

"I think it made more sense to have it in the KBU [the first year of postgraduate training] when you have something clinical to build on. Of course, you are in the clinical setting during your rotations, but you don't see patients to the same degree […] – you don't really need the skills there. So, it definitely makes more sense at the postgraduate rather than the undergraduate level." (I4, F, IM, 2-4Y)

The responsibilities of clinical work, which were more distant in their undergraduate communication skills courses, made them feel the need for communication training at this time. Furthermore, because it helped them in their work, they all valued that the training took place shortly after graduating (6-8 months) rather than later. Some stated that the timing was right for another reason – they were still open to learning communication skills:

"Well, I think that the timing of the KBU course worked really well at a point when you were a bit fresh and malleable and didn't have too many bad habits […]." (I4, F, GP, 2-4Y)

The majority thus agreed that the timing was optimal. However, as most residents that participated in the course were placed in general practice at the time of the course, many of the challenges and cases that the participants presented and worked with during the course also related to general practice. Some participants from hospital departments (for example, psychiatry and internal medicine) found that barriers to practising skills and video-recording were embedded in this particular clinical context and that cases from general practice were not always applicable to their daily hospital work.

### Experiential methods

A recurrent theme in the study related to participants' memories of, and experiences with, the experiential methods used during the course. Case-based role play, video recording and peer feedback made a notably long-lasting impression on the participants. In general, they valued these teaching methods and found that they were clearly in line with their own practice at the time:

"I think it is very useful and educational, sitting across from someone and looking them in the eye, and to try to figure out what the problem is". (I4, F, IM, 2-4Y)

Furthermore, as seen in the following quotation, role play cases based on the participants' own challenging cases, made the feedback, and suggested communicative strategies, directly applicable to their clinical practice.

"That is also what I remember. That I had brought some really concrete communication challenges that I had met in my work, and I received some input on how to handle them differently". (I4, F, IM, 2-4Y)

Despite the overall positive view on role play, a few expressed ambivalence about the method, perceiving it as intimidating while recognizing its value for learning:

"Role play is horrifying, but I understand the idea behind it". (I1, F, O, <2Y)

This ambivalence was even more clearly observed in the participants' views on using video-recording of one's own real patient encounters and receiving feedback from peers and experts. Participants found that they gained new insights into their communication through video feedback; however, some also found the method to be challenging. While a few did not mind video-recording at all, a clear majority found the method to be somewhat anxiety-provoking, albeit at the same time, perceived as a highly effective learning method. As one resident vividly expressed it:

"The part where you had to video-record yourself, it was really awful, but really good actually, both to watch the others, but also to see yourself […]. So, in retrospect, one has to say that you learn a lot from it, but I would not recommend it, mainly because it is so terrifying, but it is really good". (I3, M, GP, 2-4Y)

Despite this ambivalence, learning derived from the method was considered valuable, and all participants stated that they both learned from watching their own video-recorded consultation as well as those of their peers. A particular approach was mentioned in relation to this, namely that participants had been encouraged to bring a video-recording of a situation where they felt challenged in their communication. This was mentioned as especially valuable:

 "And for our video, I remember that there were some parts that made it fun, and it was actually better than I had thought […], probably because it was suggested that you shouldn't just bring any video, but preferably a challenging one, and several people in my group did that […]" (I2, M, GP, >4Y)

For some, sharing challenging videos, as opposed to videos where the communication had been handled highly effectively, created a valuable learning experience.

### Sharing challenges with peers

Sharing challenging situations with peers at that point in their professional life was also something they valued. One exercise they clearly remembered as useful involved sharing challenging cases, because it reminded them about not being isolated and alone when they occasionally felt professionally incompetent and inefficient.

"We started the course by sitting around a big table, and then we shared a difficult conversation we had had. And that was great, because then it became personal. It kind of created a sense of unity, and 'it's not only me who feels it's difficult. I have also experienced that, and he has experienced the same thing', […] and you could share your experiences". (I4, F, IM, 2-4Y)

### Need for continuous feedback and follow-up

Another key theme was continuous feedback and ongoing development of communication competencies. All participants expressed the need for some form of feedback on their current daily workplace communication:

I thought it was good that it was during our first year. It was very good, but one could use more later. (I1, F, GP, 2-4Y)

However, opportunities to give and receive feedback from colleagues on communication were perceived as rare. Some

had experienced local initiatives in departments or general practice where feedback on communication was put on the agenda, and they valued this feedback:

"At the department of respiratory medicine, it has been really great […]. It has been a brush-up of everything, which was needed because it was such as long time since I had anything to do with communication, so that has been great. I think it has been really good to have a follow-up." (I3, F, IM, >4Y)

However, most reported that they lacked more concrete feedback on their communication skills than they received.

"I think it is so important, and I would really like some more feedback in the department from the nurses […]. I usually ask for it a lot in the beginning when I start in a new department, and then I tire because it never happens. It would be good to get it as part of everyday practice when you are there in actual situations with the patients". (I4, F, IM, 2-4Y)

In addition to informal feedback in the workplace, participants also expressed the need for more formal mandatory follow-up training. The participants requested brush-up initiatives, tailored to their level of experience, as part of their specialist training. Exempted from this were specialties such as oncology that had already integrated formal communication into their training program. The participants stated that experience with patient care, and trust in their own clinical judgement, made them feel more relaxed and better able to think about how to use their communication skills. In their view, follow-up communication training would be worthwhile because their own level of expertise was greater:

"I think it is definitely good to have a brush-up, but it is also now that I could use it as I have seen 500 more patients, and then the ability for self-reflection is much higher because you are looking at concrete examples rather than relying on memory, I mean, you have had experiences where it ended in disaster, and you wish you had recorded them […] and as we talked about, you are just more mature, because now you know more and have a better grasp of the clinical knowledge […]" (I2, M, GP, >4Y)

All participants expressed the need for follow-up courses during specialist training to evaluate and build upon their current skills. Whilst very few would volunteer for such courses or other more informal initiatives, they would happily attend if such training was mandatory. This is witnessed in this dialogue between two participants:

"I don't get a lot of feedback [on communication], I don't think I do […] I would really like to do it more, but you know what? Every time I hear it, I think' I would love to do that, let's do it today', and then right after, I think 'but I really don't want to." (I2, M, GP, >4Y)

"But when you are forced to do it, you want to do it." (I4, GP, 2-4Y)

(I2, M, GP, >4Y) Nods.

## Discussion

Our study found that all focus group participants experienced their communication skills course during residency as a useful component in their postgraduate medical training. However, they used communication skills in different ways. Some used them as a subconscious toolkit, others very consciously and reflectively, and mainly during challenging situations that were either foreseen or occurred suddenly. In addition, participants expressed positive opinions about the fact that the course was placed at the postgraduate level when they faced the challenges of the communicative reality of medical practice and before they had developed bad habits. Teaching methods were perceived as somewhat intimidating yet of high educational benefit. Sharing challenges with peers was a valuable aspect as it prevented professional isolation. Feedback during daily practice was perceived as insufficient and rarely done.

As mentioned, we found that the participants viewed the postgraduate formal communication skills training as highly useful, and request more. This is in contrast to studies showing that such formal training was not perceived as useful.^26^ It is interesting that while all participants state that they use the communication skills learned, there are different modes of use: from full implementation to using skills only when challenged. All requested more feedback in their continuing clinical work and appreciated follow-up initiatives in the workplace. This is congruent with other studies.^5^^,^^27^ However, interestingly, some residents wished for more informal feedback in the workplace, as well as sessions with colleagues in the same specialty. Our findings raise questions about the link between the mandatory course and work-based education in the clinical departments. Residents experienced limited opportunities for sharing communicative challenges with other doctors or peers and a lack of feedback and follow-up to retain, practice, and develop communicative competencies in the clinical setting. Hence, the transfer of knowledge and skills into clinical practice can be limited or challenged by different norms, everyday routines or 'bad habits'.^28^^-^^30^ This general lack of formal training opportunities within the context of the clinical setting could explain the residents' 'need' for more mandatory external training. An implication of our findings is thus to invest more in both formal and informal postgraduate communications skills training.

Other studies have found that junior doctors stress the need for training in communication issues that are complex, challenging and relevant to their working context.^5^^,^^15^^,^^31^^,^^32^ Our findings are in line with this as they show that participants valued being asked to bring cases and videos from actual patient encounters. Most valued were challenging and difficult video-recorded patient conversations. These provided rich material for discussing, exploring, reflecting, and developing communication skills within a safe and collegial learning space. In their view, videos of encounters that were straightforward and unchallenging and where the participant performed well did not provide the same fertile learning material for professional development.  Educational frameworks, programs and assessments are often oriented towards 'best performance'. Future research should explore this aspect more thoroughly to develop new modes of learning and assessment.

Studies have shown that doctors value their undergraduate acquired communication skills more when they are at the postgraduate level.^18^ Furthermore, we have shown that doctors perceived postgraduate communication training as more relevant than undergraduate communication training as it related directly and specifically to their clinical work. This indicates that more attention should be given to postgraduate training. In addition, "early career" placement of the course, before too many 'bad habits' are embedded, is valuable. However, one problem with the timing was that general practice issues, and cases tended to dominate as most of the residents were in general practice at the time of course. It is a well-known problem that in undergraduate teaching, certain specialties, such as general practice, tend to dominate.^33^ A practical implication for medical education is thus to ensure that postgraduate communication skills training is tailored to the specific clinical contexts, including hospital settings.

We observed that participants valued sharing their communication challenges with peers, which was not an aspect of the workplace-based learning environment. The formal course provided not only communicative skills but also the opportunity to reflect on experiences and challenges with peers. This is an interesting finding in the light of studies that show that residents may feel isolated and insecure about their own professional performance and emotions.^34^^-^^36^

The study has limitations. Our findings relate to the specific context of Danish postgraduate training, and may thus be limited. However, using the Danish setting as a case study is valuable as it enables a long-term evaluation of postgraduate communication training across specialties. Methodologically, the focus group provides valuable multi-layered perspectives on a focused topic. Focus groups can, however, only say something about the topics discussed by the participants, who in our study may be people with a particular interest in communication. Others might have different opinions and perspectives.

Furthermore, not all specialities were included; for example, there were no participants from the surgical or psychiatric specialties. This prevented us from achieving full data saturation. We found repeated themes and variations, but we cannot rule out the possibility that new themes would have appeared had we conducted more focus group interviews. However, in support of our findings, the participants formal course evaluations of the short-term perceptions were generally very positive. The fact that the moderator of the focus group also was a teacher of the course is valuable. Hence, as she had in-depth knowledge, she was able to ask relevant questions. On the other hand, this could also affect participants' answers. Moreover, the median time from completing the course to conducting the interviews was 2-4 years. As such, we are not able to explore and include the perspectives of specialists or more senior doctors. Naturally, a consequence of investigating long-term retrospective perspectives is that participants' memories and experiences of other communication training situations might influence the findings. However, participants generally discussed aspects that were specifically related to the content of the course.

## Conclusions

Our study showed that doctors value early mandatory postgraduate communication skills training even years after attending the course and request similar ongoing initiatives. In addition, the timing of the training at the postgraduate level was meaningful to the doctors because this was when they faced the challenges of the communicative reality of medical practice. They all experienced that the acquired skills were useful in their ongoing clinical work, even though they used these skills in different ways.

### Acknowledgements

We want to thank the doctors who participated in our focus groups for their time and involvement. We also want to thank Signe Schlichting Matthiesen for assisting us in the research process.

### Conflict of Interest

The authors declare that they have no conflict of interest.

## Supplementary material

Supplementary file 1 Appendix. Three-day mandatory communication skills training course for residents in the Central Region, Denmark
