# Supplementary material for: Doctors' experiences of earlier mandatory postgraduate communication skills training: a qualitative study
Source: Int J Med Educ. 2022 Feb 28;13:47–55. doi: 10.5116/ijme.6213.7ee7 (PMC9017507; doi:10.5116/ijme.6213.7ee7)
Supplement: Supplementary file 1 — Appendix. Three-day mandatory communication skills training course for residents in the Central Region, Denmark [file ijme-13-47-S1.pdf]

Appendix

Three-day mandatory communication skills training course for residents in the Central Region, Denmark

Since 2004, all first-year residents have attended a mandatory three-day communication skills course focusing on doctor-patient communication that builds on existing undergraduate training. The five Danish regions organize the courses. In the Central Denmark Region, which is the context of this study, approximately 300 first-year residents complete the course each year. Residency training in communication is a subsequent part of specialty training and part of the competency-based assessment in each department. All medical specialties have objectives related to communication skills for residents to learn through daily work in the clinical setting. These objectives are intended to bridge prior communication training with clinical practice and develop the acquired work-based competencies.

Course structure

Three days: Module 1=two days (three-four weeks break with home assignments, e.g., video recording of patient conversation); Module 2=one day

Participants

Fifteen residents participate in each course. When attending the course, they are in the second part of their first-year residency and have worked as doctors for approximately 6-10 months. Most (80%) are undertaking a six months' placement in general practice, 10 % are working in psychiatry, and the last 10 % are internal medicine residents.

Content

Course content: Calgary Cambridge Observation guide, Communicating with relatives, Dealing with emotions, Breaking bad news, Lifestyle changes, Working with an interpreter, Communicating as a young doctor.

Methods

These include experientially based methods, such as:

Module 1

- Reverse role play based on participants' cases. This involves residents sharing two situations where they have experienced challenging communication situations. In small groups of five, these cases form the basis of a role play, where the case owner plays his/her own patient.
- Role-play/forum theatre. This involves large group role play, with a pre-defined case and with the
  patient played by one of the trainers.

Module 2

- Video-recorded patient consultations followed by peer and expert feedback. Here, each participant records a video of a consultation and shows it to a small group. They are instructed to bring a video where they felt most challenged in their communication.

In addition, other methods are used, such as analysis and skills spotting in video-recorded examples, short presentations, discussion, and reflection on one's own practice.

Trainers

Each course is taught by three facilitators: one with a communication background and two medical doctors. The trainers come from a variety of professional backgrounds and medical specialties: two trainers have a communication background; the rest are all medical doctors from general practice, orthopedic surgery, respiratory medicine, pediatrics, gynecology/obstetrics, psychiatry, oncology, clinical pharmacology, anesthesia, clinical genetics and otorhinolaryngology.

All trainers have taken a four-day trainers' course before becoming facilitators on the courses. All trainers participate yearly in continuous professional development initiatives and other courses such as international courses run by EACH International Association of Communication in Healthcare.

Evaluations

Evaluations of the mandatory first-year resident course, conducted immediately after the course, show overwhelming satisfaction among participants, e.g., on a 5-point Likert scale, 560-course participants from 2018-2020 have an average score of 4.64 to the question "To what degree you can use what you have learnt in everyday clinical practice?"
